# Medical Convention in Philadelphia

**Published:** 1847-03

**Authors:** 


### THE WESTERN JOURNAL.

New Series.—Vol. VII.—No. III.

LOUISVILLE, MARCH 1, 1847.

### MEDICAL CONVENTION IN PHILADELPHIA.

At the close of our last number, we made a passing reference to the approaching National Convention, in which a fear was intimated, that it would not be productive of the good which some of our brethren anticipate. Lest this should be construed into an expression of indifference or discouragement, we are led to recur to the subject.

Our apprehensions are threefold:

1. That all the schools of the United States will not send delegates, and that those who do, will not carefully discuss and set forth to those who represent them, their *desiderata;* without which there can be no adequate ground of hope, that what the convention may recommend, will be ratified by a sufficient number of our institutions to produce the desired uniformity.

2. That from the vast geographical extent of our country, and the consequent diversity of conditions under which our schools are placed, it will be difficult to establish uniformity. This will especially prove to be the case, if their representatives should not come together in a spirit of courteous and patient forbearance. If they do, much, though doubtless not all that is desirable, may be accomplished.

3. That the delegates of our medical societies will be overanxious to regulate the polity of our schools, of which most of them must necessarily know much less, and be less qualified to judge correctly, than those who have experience as teachers. Not a few of the imperfections which our medical institutions present, result from the state of society in which we live, and cannot be corrected by the convention; while others are the consequence of their number, the blame of which rests on our respective State Legislatures.

We foresee still other difficulties and embarrassments to the action of the convention, but they are of lesser magnitude, and may be obviated, provided its members shall consult together in a right spirit—that is, with a fixed determination not to part, till they have laid the foundation of an *American* profession. It is quite time that this was at least attempted. At present, there is as little intercourse and sympathy, between the physicians of the different States of the *Union* so called, as between those of the different kingdoms of Europe. All this is wrong, and should if possible be corrected. The medical profession is one throughout the world, and especially should its members in the same confederacy of States, compose one brotherhood. As a means of promoting this interesting object, and at the same time giving an impulse to medical inquiry and improvement, it is to be hoped, that the convention will not adjourn without taking measures to bring about, in some central city, a yearly meeting of physicians, surgeons, obstetricians, druggists and dentists, open to all who legitimately belong to these respective departments of the profession. Such meetings could not fail to call forth many valuable papers and reports of committees, the reading and discussion of which would be a source of both pleasure and improvement; while our physicians generally, would acquire a new impulse to observation and experimental research, and the publications of the association would elevate the character of our profession.

Our readers will recollect that the meeting in Philadelphia will be an adjournment of that held in New York in the month of May, 1846. The minutes of that meeting are now before us, and we observe that several committees were appointed, to make reports on the organization of a national association—on the primary education of students of medicine—on a higher standard of graduation—on state boards of examiners for the degree of M.D., instead of its being done by the professors—finally, on a uniform system of medical ethics. All these are important topics, which it is hoped the different committees will thoroughly investigate. There was also a committee, of which

Prof. Knight, of New Haven, was the chairman, appointed to prepare and publish an address to the physicians of the United States, explaining to them the objects of the proposed meeting in Philadelphia, and urging their coöperation. From their address we make the following extract:

"It remains to be seen whether these desirable objects shall be accomplished. It is obvious that this can be done only by general and united exertion. All partial or divided action will be unavailing. It is equally true, that whatever is done must be accomplished by medical men themselves. In some countries, the interests of the medical profession, like others of great importance to the community, are regulated, sustained and protected by public law. In this country, no general law, even if it were desirable, can ever exist. In many of the States, no laws upon this subject have ever been enacted; in several, where they have formerly existed, they have been repealed; and in those where they still remain, there is a general complaint of their inefficiency. In this state of things, the only resource which remains is for medical men to establish and enforce among themselves such regulations as shall purify and elevate their own body, and thus more fully command the respect and confidence of their fellow men. The proposed association, if it becomes general, may be the means of accomplishing much of this good work. The opinions of such a body of the most respectable members of the profession, enjoying the confidence of their brethren and of the public, freely expressed after full consultation and careful deliberation, although not clothed with the authority of law, will still command respect, and, for the most part, compliance. A public opinion in regard to the subjects decided upon will be created, which will be more controlling than law. It is by creating and sustainihg a sound and healthy public opinion, that the association will prove most beneficial."

We shall only add, that the University of Louisville has determined to send a delegation, consisting of Professors Drake, Cobb, and Yandell.

